# Personalia

**Published:** 1914-04-18

**Authors:** 


### Personalia.

Dr. F. W. Mott, the well-known director of the London County Council's Pathological Laboratory, is to have conferred upon him by the University of Edinburgh the honorary degree of Doctor of Laws.

Colonel R. J. Cooper, Mrs. Curteis, Mr. George Dew, Mrs. Dunn-Gardner, Mr. T. Hunter, Mr. S. Joyce Thomas, and Lord Windsor have been appointed as the London County Council's visiting sub-committee for Banstead Mental Hospital.

Mr. T. Chapman, Captain the Hon. A. C. S. Chichester, Mr. E. G. Easton, Mr. W. C. Johnson, Mr. Henry Mills, Miss Ida Samuel, Mr. E. Tomkins, and Sir Edward White will form the London County Council's visiting committee for Colney Hatch Mental Hospital during the coming year.

Lord Cheylesmore has been appointed as the London County Council's representative on the Chapter of the College of St. Katharine-by-the-Tower, which is being formed in connection with the reorganisation of St. Katharine's Hospital.

